# Supplementary material for: Deletion of wfs1 Impairs Oligodendrocyte Precursor Cells Dorsal Distribution and Myelination Through the wfs1-hmgcs1 Axis in Zebrafish
Source: Biology (Basel). 2026 Aug 16;15(16):1402. doi: 10.3390/biology15161402 (PMC13510151; doi:10.3390/biology15161402)
Supplement: Supplementary file 1 [file biology-15-01402-s001.zip › File S1-Transcriptome sequencing data and differentially expressed genes (DEGs) in wfs1-- zebrafish at 5 dpf/2.KEGG/wfs1KOvsWT/GSEA/WT/OTHER_GLYCAN_DEGRADATION/OTHER_GLYCAN_DEGRADATION(DRE00511).html]

Details for gene set OTHER\_GLYCAN\_DEGRADATION(DRE00511)[GSEA]

|  || Dataset | deg.sample |
| Phenotype | sample.cls |
| Upregulated in class | WT |
| GeneSet | OTHER\_GLYCAN\_DEGRADATION(DRE00511) |
| Enrichment Score (ES) | -0.5501884 |
| Normalized Enrichment Score (NES) | -1.5339351 |
| Nominal p-value | 0.031578947 |
| FDR q-value | 1.0 |
| FWER p-Value | 0.557 |
Table: GSEA Results Summary

  

Fig 1: Enrichment plot: OTHER\_GLYCAN\_DEGRADATION(DRE00511)      
 Profile of the Running ES Score & Positions of GeneSet Members on the Rank Ordered List

  

| PROBE | DESCRIPTION (from dataset) | GENE SYMBOL | GENE\_TITLE | RANK IN GENE LIST | RANK METRIC SCORE | RUNNING ES | CORE ENRICHMENT || 1 | 566517 | aga |  |  | 969 | 2.133 | 0.0622 | No |
| 2 | 559072 | gba |  |  | 3533 | 0.923 | 0.0218 | No |
| 3 | 565372 | hexdc |  |  | 7210 | 0.473 | -0.0731 | No |
| 4 | 548343 | glb1 |  |  | 7702 | 0.440 | -0.0694 | No |
| 5 | 541519 | man2b1 |  |  | 10713 | 0.260 | -0.1526 | No |
| 6 | 550460 | hexa |  |  | 11206 | 0.233 | -0.1579 | No |
| 7 | 323613 | hexb |  |  | 12362 | 0.171 | -0.1868 | No |
| 8 | 100000684 | neu3.5 |  |  | 14952 | 0.049 | -0.2659 | No |
| 9 | 561239 | engase |  |  | 16009 | -0.002 | -0.2989 | No |
| 10 | 445487 | fuca2 |  |  | 16034 | -0.003 | -0.2996 | No |
| 11 | 569595 | man2c1 |  |  | 16388 | -0.014 | -0.3101 | No |
| 12 | 559240 | gba2 |  |  | 16594 | -0.020 | -0.3156 | No |
| 13 | 393128 | manba |  |  | 16754 | -0.024 | -0.3196 | No |
| 14 | 566517\_1 | aga |  |  | 22122 | -0.274 | -0.4761 | No |
| 15 | 555206\_1 | neu3.3 |  |  | 23654 | -0.371 | -0.5081 | No |
| 16 | 559850 | neu1 |  |  | 23773 | -0.378 | -0.4954 | No |
| 17 | 553569 | neu4 |  |  | 25521 | -0.486 | -0.5291 | Yes |
| 18 | 335494 | fuca1.1 |  |  | 26131 | -0.540 | -0.5248 | Yes |
| 19 | 793046\_1 | neu3.4 |  |  | 26386 | -0.566 | -0.5082 | Yes |
| 20 | 445165 | fuca1.2 |  |  | 26581 | -0.586 | -0.4888 | Yes |
| 21 | 767799 | zgc:153169 |  |  | 26609 | -0.589 | -0.4641 | Yes |
| 22 | 100003570 | man2b2 |  |  | 27071 | -0.637 | -0.4510 | Yes |
| 23 | 565372\_1 | hexdc |  |  | 27474 | -0.670 | -0.4345 | Yes |
| 24 | 780844 | neu3.1 |  |  | 27740 | -0.708 | -0.4121 | Yes |
| 25 | 541364 | zgc:113333 |  |  | 29912 | -1.206 | -0.4279 | Yes |
| 26 | 100000684\_1 | neu3.5 |  |  | 30984 | -1.951 | -0.3769 | Yes |
| 27 | 445250 | neu3.2 |  |  | 31793 | -4.548 | -0.2049 | Yes |
| 28 | 445250\_1 | neu3.2 |  |  | 31824 | -4.790 | 0.0021 | Yes |
Table: GSEA details [plain text format]

  

Fig 2: OTHER\_GLYCAN\_DEGRADATION(DRE00511)      
 Blue-Pink O' Gram in the Space of the Analyzed GeneSet

  

Fig 3: OTHER\_GLYCAN\_DEGRADATION(DRE00511): Random ES distribution      
 Gene set null distribution of ES for **OTHER\_GLYCAN\_DEGRADATION(DRE00511)**
